# Preclinical characterization of amubarvimab and romlusevimab, a pair of non-competing neutralizing monoclonal antibody cocktail, against SARS-CoV-2

**DOI:** 10.3389/fimmu.2022.980435

**Published:** 2022-09-14

**Authors:** Yun Ji, Qi Zhang, Lin Cheng, Jiwan Ge, Ruoke Wang, Mengqi Fang, Eric M. Mucker, Peng Chen, Ji Ma, Rui Zhang, Chunming Li, Holly Hammond, Lauren Baracco, Michael Holbrook, Matthew Frieman, Zheng Zhang, Xinquan Wang, Jay W. Hooper, Linqi Zhang, Qing Zhu

**Affiliations:** ^1^ Brii Biosciences Inc., Durham, NC, United States; ^2^ Center for Global Health and Infectious Diseases, Comprehensive AIDS Research Center, Department of Basic Medical Sciences, School of Medicine, Tsinghua University, Beijing, China; ^3^ Institute for Hepatology, National Clinical Research Center for Infectious Disease, Shenzhen Third People’s Hospital, Shenzhen, China; ^4^ The Ministry of Education Key Laboratory of Protein Science, Beijing Advanced Innovation Center for Structural Biology, Beijing Frontier Research Center for Biological Structure, Collaborative Innovation Center for Biotherapy, School of Life Sciences, Tsinghua University, Beijing, China; ^5^ U.S. Army Medical Research Institute of Infectious Diseases (USAMRIID), Fort Detrick, MD, United States; ^6^ Brii Biosciences Inc., Beijing, China; ^7^ Center for Pathogen Research, Department of Microbiology and Immunology, University of Maryland School of Medicine, Baltimore, MD, United States; ^8^ Integrated Research Facility, Division of Clinical Research, National Institute of Allergy and Infectious Diseases (NIAID), Fort Detrick, MD, United States

**Keywords:** monoclonal antibody (mAb), severe acute respiratory syndrome coronavirus 2 (SARS-CoV-2), receptor binding domain (RBD), M252Y/S254T/T256E (YTE), amubarvimab (BRII-196), romlusevimab (BRII-198), variant of concern (VOC), half-maximal inhibitory concentration (IC_50_)

## Abstract

Monoclonal antibodies (mAbs) targeting the severe acute respiratory syndrome coronavirus 2 (SARS-CoV-2) spike protein have demonstrated clinical efficacy in preventing or treating coronavirus disease 2019 (COVID-19), resulting in the emergency use authorization (EUA) for several SARS-CoV-2 targeting mAb by regulatory authority. However, the continuous virus evolution requires diverse mAb options to combat variants. Here we describe two fully human mAbs, amubarvimab (BRII-196) and romlusevimab (BRII-198) that bind to non-competing epitopes on the receptor binding domain (RBD) of spike protein and effectively neutralize SARS-CoV-2 variants. A YTE modification was introduced to the fragment crystallizable (Fc) region of both mAbs to prolong serum half-life and reduce effector function. The amubarvimab and romlusevimab combination retained activity against most mutations associated with reduced susceptibility to previously authorized mAbs and against variants containing amino acid substitutions in their epitope regions. Consistently, the combination of amubarvimab and romlusevimab effectively neutralized a wide range of viruses including most variants of concern and interest *in vitro*. In a Syrian golden hamster model of SARS-CoV-2 infection, animals receiving combination of amubarvimab and romlusevimab either pre- or post-infection demonstrated less weight loss, significantly decreased viral load in the lungs, and reduced lung pathology compared to controls. These preclinical findings support their development as an antibody cocktail therapeutic option against COVID-19 in the clinic.

## Introduction

The coronavirus disease 2019 (COVID-19) pandemic caused by severe acute respiratory syndrome coronavirus 2 (SARS-CoV-2) continues to be a tremendous challenge to healthcare system that has resulted in nearly 522 million confirmed cases and over 6 million deaths worldwide as of 22 May 2022 (1). While several SARS-CoV-2 vaccines have demonstrated efficacy at reducing severe disease and death, the effective control of COVID-19 remains vulnerable as steeply increased infection continued to be found not only among unvaccinated but also vaccinated individuals. Such continuous and wide-spread infection and breakthrough infection are expected to generate and select variants with increased transmissibility and immune evasion, rendering many therapeutics and vaccines less effective. Thus, additional medical countermeasures are needed to reduce COVID-19 induced morbidity and mortality.

Neutralizing monoclonal antibodies (mAbs) represent an important therapeutic option in the prevention and treatment of known and emerging infectious diseases (2, 3). SARS-CoV-2 uses the angiotensin-converting enzyme 2 (ACE2) to enter cells *via* interaction with the receptor binding domain (RBD) of its spike protein (4). Therefore, several RBD-targeting mAbs including casirivimab/imdevimab, bamlanivimab/etesevimab, sotrovimab were selected for clinical development (5–8). The first 4 mAbs have epitopes fully or partially overlapping with the receptor-binding motif (RBM) on the RBD thereby blocking viral entry by preventing ACE2 from binding to RBM, whereas non-RBM mAbs such as sotrovimab appears to block viral infection by sterically interfering the viral membrane fusion after ACE2 engagement with RBM (9). Besides different mechanisms of action (MoA) exerted by these antibodies, various modifications to their fragment crystallizable (Fc) were also employed to prolong their half-lives and alleviate potential antibody-dependent enhancement of infection *in vivo*. For example, Fc effector function was abolished by the L234A/L235A (LALA) modification (etesevimab) whereas half-life was extended with the M248L/N434S (LS) modification (sotrovimab). Other clinical antibodies, however, were developed as wild-type IgG1 (casirivimab/imdevimab, bamlanivimab) (10). It is gratifying to see that all these clinical mAbs demonstrated efficacy in reducing viral burden, hospitalization, and death among mild to moderate COVID-19 compared to untreated placebo controls. Many of these antibodies have been authorized under emergency use authorization (EUA) for the treatment and/or prevention of severe disease in outpatients with mild to moderate COVID-19 who are at high risk of clinical progression (11–14). However, like other infectious organisms, SARS-CoV-2 continues to mutate over time, generating antigenically distinct variants that are responsible for multiple waves of rapidly spread infection worldwide. Some variants are resistant to these mAbs, resulting in substantial reduction or completely loss of their clinical efficacy for the treatment and prevention of SARS-CoV-2 infection (15). In particular, the recent emergence of Omicron subvariants is the most distinctive in antigenic properties and capable of escaping from many therapeutic antibodies (15–17). FDA has recently revised authorizations for casirivimab/imdevimab, bamlanivimab/etesevimab, and sotrovimab in geographic regions where infection is likely to have been caused by a non-susceptible SARS-CoV-2 variant. Thus, antibody therapeutics capable of overcoming viral escape and mitigate the impact of variants are highly desirable.

Amubarvimab (BRII-196) and romlusevimab (BRII-198) are human IgG1 mAbs derived from their respective precursor antibodies, P2C-1F11 and P2B-1G5, which were isolated directly from B cells of a convalesced COVID-19 patient showing specific activity against wild-type SARS-CoV-2 and no cross-reactivity against other coronaviruses (18). Amubarvimab binds to two regions spanning amino acids 453-505 in the RBM and amino acids residues 403-421 in the core region of RBD. Among its predicted contact residues, 11 of 23 overlap with the ACE2 binding sites on SARS-CoV-2 RBD, providing the structural basis for amubarvimab in competing with ACE2 for binding to the RBD and blocking subsequent virus entry (19). In addition, the Fc region of amubarvimab and romlusevimab was engineered with a triple-amino-acid (M252Y/S254T/T256E [YTE]) substitution to allow an extended half-life to potentially prolong the treatment window and reduce effector functions (20–23).

Here we characterized the antiviral activity and resistance profile of amubarvimab and romlusevimab *in vitro* and *in vivo*. These two mAbs simultaneously bind to the distinct non-competing epitopes on the RBD of the spike protein, showing distinctive Fc receptor binding features which led to their extended half-life and reduced effector functions. Furthermore, the combination of amubarvimab and romlusevimab can effectively neutralize several SARS-CoV-2 live virus variants *in vitro* as well as most emerging variants of concern/interest (VOC/VOI) and variants that confer resistance to previously authorized mAbs in pseudotyped virus assays. Phenotypic analysis of the predicted contact residues in the epitope region of both antibodies indicates the potential for a high barrier to resistance due to the complementary effects of the cocktail strategy. Additionally, data derived from the Syrian golden hamster model demonstrates anti-viral efficacy *in vivo*. Recently, amubarvimab and romlusevimab in combination demonstrated efficacy at reducing risk of progression to severe disease among high risk outpatients with mild to moderate COVID-19 at a period of time with multiple circulating variants (24). Taken together, these data indicate that amubarvimab and romlusevimab are valuable antibody cocktail for the treatment of COVID-19.

## Materials and methods

### Antibody binding and competition measured by surface plasmon resonance

Measurement of equilibrium dissociation constants between RBD and antibodies: The CM5 sensor chip was activated with 400 mM EDC and 100 mM NHS (prepared immediately before use) for 420 s at a flow rate of 10 μL/min with the mixture. Thirty μg/mL of goat anti-human IgG Fc antibody in 10 mM NaAc (pH 4.5) was then injected to channels for 420 s at a flow rate of 10 μL/min. The chip was deactivated by 1 M ethanolamine-HCl (GE) for 420 s at a flow rate of 10 μL/min. Antibodies in running buffer 1×HBS-EP+ (0.01 M HEPES, 0.15 M NaCl, 3 mM EDTA, 0.05% surfactant P20, pH 7.4) were captured onto Fc2 *via* anti-human IgG Fc at a flow rate of 10 μL/min. Six concentrations of RBD (1.5625, 3.125, 6.25, 12.5, 25 and 50 nM) in running buffer were injected into Fc1 and Fc2 at a flow rate of 30 μL/min for an association phase of 120 s, followed by 240 s dissociation. 10 mM glycine (pH 1.5) as regeneration buffer was injected to flow cells for 30 s twice at a flow rate of 30 μL/min following every dissociation phase.

Competition of antibodies for binding to RBD measured by SPR: To determine the competitive binding of antibodies to RBD of spike protein, SARS-CoV-2 RBD was immobilized to a CM5 sensor chip *via* amine groups in 10 mM sodium acetate buffer (pH 5.0) for a final RU around 250. Next, P2C-1F11 and P2B-1G5 were sequentially injected and monitored for binding activity to determine whether the two mAbs recognized separate or closely situated epitopes (18).

Antibody competition with ACE2 for binding to RBD measured by SPR: The spike protein (20 µg/ml) was captured on the anti-His antibody biosensor for 30 s and stabilized for 30 s. The serially diluted antibodies (0.781-200 nM) were incubated with the sensors for 120 s to allow antibody and spike protein binding. Immediately, the sensors were dipped in the ACE2 solution (300 nM) for 120 s to record the response signal. For analysis of the half-maximal inhibition concentration (IC_50_), the dose-blocking curves were plotted and the blocking IC_50_ values were calculated by nonlinear fit using GraphPad Prism 9 software.

Antibody binding affinity to neonatal Fc receptor (FcRn) measured by SPR: mAb was immobilized by amine coupling to a sensor chip. An 8-fold dilution series of human FcRn was prepared at 46.875, 93.75, 187.5, 375, 750, 1500, 3000 and 6000 nM in pH 6.0 1x PBST buffer, then individually injected over the mAb surface and the binding responses were recorded.

Antibody binding affinity to FcγR measured by SPR: The activator was prepared by mixing 400 mM N-ethyl-N’-(dimethylaminopropyl) carbodiimide and 100 mM N-hydroxysuccinimide immediately prior to injection. The CM5 sensor chip was activated for 420 s at a flow rate of 10 μL/min with the mixture. Thirty μg/mL of THE™ His tag antibody in 10 mM NaAc (pH 4.5) was then injected for 400 s at a flow rate of 30 μL/min. The chip was deactivated by 1 M ethanolamine-HCl (GE) for 420 s at a flow rate of 10 μL/min. Optimal concentration of FcγRs in running buffer (1×HBS-EP+) was injected to Fc2 at a flow rate of 10 μL/min. Eight concentrations of mAbs and running buffer were injected to Fc1-Fc2 at a flow rate of 30 μL/min for optimized duration of association and dissociation phases. Ten mM glycine (pH 1.5) as regeneration buffer was injected to the sensor chip surface for 30 s twice at a flow rate of 10 μL/min following every dissociation phase. The chip was next regenerated with 10 mM glycine (pH 1.5).

### *In vitro* escape mutants screening

Generation of replication-competent recombinant vesicular stomatitis virus (VSV)-SARS-CoV-2: The recombinant VSV-SARS-CoV-2 virus was generated using the method described previously by co-transfection of pVSV-N, pVSV-P, pVSV-L, and pVSV-SARS-CoV-2 S-eGFP, and a recombinant vaccinia virus expressing T7 polymerase in BHK-21 cells. After 48 h incubation, the cells were frozen/thawed three times. The supernatants were added to fresh Vero E6 cells for virus recovery before centrifugation at 2000g for 10 min. The recovered recombinant VSV-SARS-CoV-2 virus was then propagated in Vero E6 cells at 37°C until the development of cytopathic effect (CPE) and stored at -80°C. Virus titers were determined by the number of foci forming units (FFU) after 24 h infection. In brief, Vero E6 cells were inoculated in 96-well plates (~ 2 × 10^4^ per well) a day before viral inoculation. Serial diluted virus stock was added to 96-well plates at 80-90% confluency of Vero E6 cells in DMEM media supplemented with 2% FBS and 20 mM NH_4_Cl. The cells were incubated for 24 h at 28°C. The FFU were detected by eGFP signals of infected Vero E6 cells with Opera Phenix.

Antibody escape studies: Antibodies were serially diluted 5-fold starting at 25 µg/mL for the first round of selection. The cocktail of amubarvimab and romlusevimab contained equal amounts of each antibody and the concentration used for selection represents the total concentration of the two antibodies. A total of 1 × 10^5^ FFU of the VSV-SARS-CoV-2 virus was added to each dilution and incubated at 37°C for 30 min. After incubation, the mixture was added to 1 × 10^5^ Vero E6 cells and incubated for 4 days in 24-well plates. Cells were screened for virus replication by monitoring for fluorescent foci. Supernatants and RNA were collected from wells under the highest concentration antibody selection with detectable viral replication measured by fluorescent units using the Opera Phenix. This is P1 supernatant.

For a second round of selection, 50 μL of the P1 supernatant was mixed with 50 μL DMEM with 2% FBS and transferred into each well of a 48-well plate that contained freshly prepared Vero E6 cells with increasing antibody concentrations ranging from 0.008-250 µg/mL. The plate was incubated for 4 days. Fluorescent units were quantitated using Opera Phenix and exported values were analyzed. RNA was extracted from the well containing the highest antibody concentration with detectable viral replication. The RNA was sequenced from both passages to identify escape mutants.

Sequence analysis: The total RNA in the supernatant of GFP positive wells with the highest antibody concentration was extracted and reverse transcribed into cDNA. The RBD gene fragment was amplified by PCR using the following primers: Forward: 5’ CACGTGTGATCAGATATCGCGGCCGCGTTCCCAAACATCACAAAC 3’,

Reverse: 5’ TAGAAGGCACAGCAGATCTGGATCCACTCGGTGAGCACGCCTG 3’. The amplified PCR product was cloned into the PVRC8400 vector and transformed into bacteria. DNA minipreps from twenty bacterial colonies were sequenced in each condition.

### Crystallization and structure determination

The SARS-CoV-2 RBD and the fragment antigen-binding (Fab) fragments of BRII-196 and BRII-198 were mixed at a molar ratio of 1:1:1, incubated for 2 h at 4 °C and were further purified by gel-filtration chromatography. The purified complex concentrated to approximately 10 mg/mL in HBS buffer (10 mM HEPES, pH 7.2, 150 mM NaCl) was used for crystallization. The screening trials were performed at 18 °C using the sitting-drop vapor-diffusion method by mixing 0.2 μL of protein with 0.2 μL of reservoir solution. Crystals were successfully obtained in 0.2 M NaCl, 0.1 M MES, pH 6.0, 20% PEG 2000 MME. Crystals were harvested, soaked briefly in mother liquid with 20% glycerol, and flash-frozen in liquid nitrogen. Diffraction data were collected at the BL17U beam line of the Shanghai Synchrotron Research Facility (SSRF). Diffraction data were auto processed with aquarium pipeline.

The structure was determined by the molecular replacement method in CCP4 suite. The search models were the SARS-CoV-2 RBD structure (PDB ID: 6M0J) and the structures of the variable domain of the heavy and light chains available in the PDB with the highest sequence identities. Subsequent model building and refinement were performed using COOT and PHENIX, respectively. Final Ramachandran statistics: 93.86% favored, 5.32% allowed and 0.82% outliers for the final structure. All structural figures were generated using PyMOL.

#### Production of pseudoviruses bearing envelopes of SARS-CoV-2 wild-type and variants

The human immunodeficiency virus (HIV) or murine leukemia virus (MLV)-based vectors carrying SARS-CoV-2 spike protein were constructed and co-transfected into pseudoviral particle-producing cells to generate pseudovirus variants that contain single mutation in the RBD or all amino acid mutations in the spike protein of representative SARS-CoV-2 lineages. The variant B.1.1.7 (Alpha) was constructed with total of 9 mutations including 69-70del, 144del, N501Y, A570D, D614G, P681H, T716I, S982A, and D1118H. The variant B.1.351 (Beta) was constructed with 9 mutations including L18F, D80A, D215G, 242-244del, K417N, E484K, N501Y, D614G, and A701V. The variant B.1.1.248/P.1 (Gamma) was constructed with 12 mutations including L18F, T20N, P26S, D138Y, R190S, K417T, E484K, N501Y, D614G, H655Y, T1027I, and V1176F. The variant B.1.427/429 (Epsilon) was constructed with 4 mutations including S13I, W152C, L452R, and D614G. The variant B.1.526 (Iota) was constructed with 6 mutations including L5F, T95I, D253G, E484K, D614G, and A701V. The variant B.1.617.1 (Kappa) was constructed with 8 mutations including T95I, G142D, E154K, L452R, E484Q, D614G, P681R, and Q1071H. The variant B.1.617.2 (Delta) was constructed with 9 mutations including T19R, G142D, 156-157del, R158G, L452R, T478K, D614G, P681R, and D950N. The variant B.1.525 (Eta) was constructed with 8 mutations including Q52R, A67V, H69-V70del, Y144del, E484K, D614G, Q677H, and F888L. The variant C.37 (Lambda) was constructed with 8 mutations including G75V, T76I, R246-D252del, D253N, L452Q, F490S, D614G, and T859N. The variant B.1.621 (Mu) was constructed with 9 mutations including T95I, Y144S, Y145N, R346K, E484K, N501Y, D614G, P681H, and D950N. The B.1.617.2 sub-lineage AY.1/AY.2 (Delta+) was constructed with total of 13 mutations including T19R, T95I, G142D, E156G, F157del, R158del, W258L, K417N, L452R, T478K, D614G, P681R, and D950N. The B.1.617.2 (Delta) sub-lineage AY.4.2 variant was constructed with total of 14 mutations including T19R, T95I, G142D, Y145H, E156G, F157del, R158del, A222V, L452R, K458R, T478K, D614G, P681R, and D950N. The Omicron BA.1 variant was constructed with total of 32 mutations including A67V, 69-70del, T95I, G142D/143-145del, 211del/L212I, ins214EPE, G339D, S371L, S373P, S375F, K417N, N440K, G446S, S477N, T478K, E484A, Q493R, G496S, Q498R, N501Y, Y505H, T547K, D614G, H655Y, N679K, P681H, N764K, D796Y, N856K, Q954H, N969K, and L981F. The BA.1.1 (Omicron+R346K) was constructed with total of 33 mutations including A67V, 69-70del, T95I, G142D/143-145del, 211del/L212I, ins214EPE, G339D, R346K, S371L, S373P, S375F, K417N, N440K, G446S, S477N, T478K, E484A, Q493R, G496S, Q498R, N501Y, Y505H, T547K, D614G, H655Y, N679K, P681H, N764K, D796Y, N856K, Q954H, N969K, and L981F. The BA.2 was constructed with total of 29 mutations including T19I, L24-26del, A27S, G142D, V213G, G339D, S371F, S373P, S375F, T376A, D405N, R408S, K417N, N440K, S477N, T478K, E484A, Q493R, Q498R, N501Y, Y505H, D614G, H655Y, N679K, P681H, N764K, D796Y, Q954H, and N969K. The BA.2.12.1 was constructed with total of 31 mutations including T19I, Δ24-26, A27S, G142D, V213G, G339D, S371F, S373P, S375F, T376A, D405N, R408S, K417N, N440K, L452Q, S477N, T478K, E484A, Q493R, Q498R, N501Y, Y505H, D614G, H655Y, N679K, P681H, S704L, N764K, D796Y, Q954H, and N969K. The BA.3 was constructed with total of 28 mutations including A67V, 69-70del, T95I, G142D/143-145del, 211del/L212I, G339D, S371F, S373P, S375F, D405N, K417N, N440K, G446S, S477N, T478K, E484A, Q493R, Q498R, N501Y, Y505H, D614G, H655Y, N679K, P681H, N764K, D796Y, Q954H, and Q992H. BA.4/5 was constructed with total of 31 mutations including T19I, Δ24-26, A27S, Δ69-70, G142D, V213G, G339D, S371F, S373P, S375F, T376A, D405N, R408S, K417N, N440K, L452R, S477N, T478K, E484A, F486V, Q498R, N501Y, Y505H, D614G, H655Y, N679K, P681H, N764K, D796Y, Q954H, and N969K.

#### Microneutralization assays of pseudotyped variants

Plasmids pCMVΔR8.2, pHR’CMVLuc and expressing plasmids encoding the wild-type or mutant spikes (codon optimized) were co-transfected into 293T cells to produce HIV-based pseudovirus variants that contain single mutation in the RBD or all mutant residues in the spike of representative SARS-CoV-2 lineages. The pseudovirus variants were evaluated in the microneutralization assay using 293T cells transduced with ACE2 and TMPRSS2 genes for stable expression (BEI Resources NR-55293), as previously described (25). Briefly, pseudoviruses with titers of approximately 10^6^ relative light unit (RLU)/ml of luciferase activity were incubated with antibodies for one hour at 37°C. Pseudovirus and antibody mixtures (100 μl) were then inoculated onto 96-well plates that were seeded with 3.0 x 10^4^ cells/well one day prior to infection. Pseudovirus infectivity was scored 48 hours later by luciferase activity. The antibody dilution or mAb concentration causing a 50% reduction of RLU compared to control (ID_50_ or IC_50_, respectively) were reported as the neutralizing antibody titers. Titers were calculated using a nonlinear regression curve fit with GraphPad Prism software. The mean 50% reduction of RLU compared to control from at least two independent experiments, each with intra-assay duplicate, was reported as the final titer or IC_50_.

Alternatively, SARS-CoV-2 wildtype or mutant pseudoviruses were generated by co-transfection of HIV backbones expressing firefly luciferase (pNL43R-E-luciferase) and pcDNA3.1 (Invitrogen) expressing the respective spike proteins into HEK-293T cells. Viral supernatants were collected 48 h later and measured the infectivity by luciferase activity in relative light units (Bright-Glo Luciferase Assay Vector System, Promega Biosciences). Neutralization assays were performed by incubating 1000 TCID_50_ pseudoviruses with serial dilutions of purified mAbs at 37°C for 1 h. HeLa-ACE2 cells (approximately 1.5 × 10^4^ per well) were added in duplicate to the virus-antibody mixture. Half-maximal inhibitory concentrations (IC_50_) of the evaluated mAbs were determined by luciferase activity measured 48 h after exposure to virus-antibody mixture using GraphPad Prism 9 software (18).

In addition, SARS-CoV-2 pseudovirus encoding spike protein (614G) or spike proteins containing the respective point mutation, or all mutations was generated by co-transfection of murine leukemia virus backbone pCMV-MLVgag-pol, pTG-Luc and pcDNA3.1 expressing the respective spike proteins into HEK-293T cells. To enhance the incorporation, the C-terminal 19 residues in the cytoplasmic tail of the SARS-CoV-2 S protein were deleted. Viral supernatants were collected 48 h later and measured the infectivity by luciferase activity using Firefly Luciferase Assay Kit (Codex BioSolutions). To prepare for infection, 7.5 x 10^3^ HEK293 cells, which stably express full-length human ACE2, were plated into a 384-well white-clear plate coated with poly-D-Lysine in 15 µl culture medium. On the day 2, 12.5 µl of SARS-CoV-2 MLV pseudoviruses were mixed with 5 µl of each mAb at a serial of concentrations and incubated at 37°C for 1 hour. After removing the medium supernatant in each well, 17.5 μl of individual mAb–virus mixture was added. The plate was centrifuged at 54g for 15 min at 4°C and an additional 7.5 μl of culture medium was then added. The total final volume in each well was 25 μl. The cells were then incubated at 37°C for 42 h. Luciferase activities were measured with Firefly Luciferase Assay Kit. IC_50_ values were calculated based on curve fitting in GraphPad Prism 9 software. All data were derived from at least two independent experiments (26).

### Microneutralization assays of live SARS-CoV-2 isolates

The live viruses tested in the assay were sequenced and compared with consensus sequences. The B.1.1.7-CA (hCoV-19/USA/CA_CDC_5574/2020 (BEI Cat#NR-54011) contained total of 10 mutations including 69-70del, N74K, 144del, N501Y, A570D, D614G, P681H, T716I, S982A, and D1118H. The B.1.1.7-PHE (hCoV-19/England/204820464/2020 (BEI Cat# NR-54000) contained total of 9 mutations including 69-70del, 144del, N501Y, A570D, D614G, P681H, T716I, S982A, and D1118H. The B.1.351 (hCoV-19/South Africa/KRISP-K005325/2020 (BEI Cat#NR-54009) contained 10 mutations including L18F, D80A, D215G, 242-244del, E484K, N501Y, D614G, Q677H, R682W, and A701V. This assay was performed by incubating a fixed volume of virus (0.5 multiplicity of infection (MOI)) with the mAbs for 1 hour at 37°C prior to adding to Vero E6 cells plated in 96-well plates. Following addition to Vero E6 cells, the virus was allowed to infect the cells and propagate for 24 hours at 37°C, 5% CO_2_, at which time the cells were fixed with neutral buffered formalin. Following fixation, the cells were permeabilized with radiolabeled immunoprecipitation buffer (RIPA) buffer and probed with a SARS-CoV-2 nucleoprotein-specific rabbit primary antibody (Sino Biological, Wayne, PA, #40143-R001) followed by an Alexa647-conjugated secondary antibody (Life Technologies, San Diego, CA, #A21245). Cells were counterstained with Hoechst nuclear stain (Life Technologies, #H3570). Cells in four fields per well were counted with each field containing at least 1000 cells, with four wells per dilution for each test sample. Plates were read and quantified using an Operetta high content imaging system (PerkinElmer, Waltham, MA). Antibodies were screened using a 2-fold serial 12-step dilution. The lower limit of detection was either 1:20 or 1:40 depending upon the dilution series. Assays were controlled using a spike protein specific antibody as a positive control in addition to virus-only and uninfected cell controls.

Alternatively, neutralization activity of mAbs against live virus using focus reduction neutralization test (FRNT) was performed in a certified Biosafety level 3 laboratory. The live viruses tested in the assay were sequenced and compared with consensus sequences. Wild-type live SARS-CoV-2 is a clinical isolate (Beta/Shenzhen/SZTH-003/2020, EPI_ISL_406594 at GISAID) previously obtained and subsequently expanded from a nasopharyngeal swab of an infected patient. The variant B.1.351 (GDPCC-nCoV84 strain, Accession No. GWHBDSE01000000 at the Genome Warehouse in National Genomics Data Center) was obtained from Guangdong Provincial Center for Disease Control and Prevention, Guangdong Center for Human Pathogen Culture Collection (GDPCC) containing 9 mutations including L18F, D80A, D215G, 242-244del, K417N, E484K, N501Y, D614G, and A701V. The variant B.1.617.2 is a clinical isolate (SZTH012 strain, Accession No. GWHBFWZ01000000 at the Genome Warehouse in National Genomics Data Center) containing 9 mutations including T19R, G142D, 156-157del, R158G, L452R, T478K, D614G, P681R, and D950N. Serial dilutions of mAbs were conducted, mixed with the same volume (1:1) of 100 FFU SARS-CoV-2 in 96-well microwell plates and incubated for 1 h at 37°C. Mixtures were then transferred to 96-well plates seeded with Vero E6 cells and allowed to absorb for 1 h at 37°C. Inoculums were then removed before adding the overlay media (100 μl opti-MEM containing 1.6% Carboxymethylcellulose, CMC). The plates were then incubated at 37°C for 24 h. Next, overlays were removed, and cells were fixed with 4% paraformaldehyde solution for 30 min. Cells were permeabilized with 0.2% Triton X-100, washed with PBS twice and incubated with cross-reactive rabbit anti-SARS-CoV-N IgG (Sino Biological, Inc) for 1 h at room temperature before adding HRP-conjugated goat anti-rabbit IgG (H+L) antibody (TransGen Biotech). Cells were further incubated at room temperature. The reactions were developed with KPL TrueBlue Peroxidase substrates (Seracare Life Sciences Inc). The numbers of SARS-CoV-2 foci were calculated using an ELISpot reader (Cellular Technology Ltd).

In addition, neutralization activity of mAbs against live virus was performed in a certified Biosafety level 3 laboratory. The live viruses tested in the assay including wild-type SARS-CoV-2 WA1/2020 (CDC): hCoV-19/USA/WA1; BA.1: hCoV-19/USA/GA-EHC-2811C/2021; BA.1.1: hCoV-19/USA/HI-CDC-4359259-001/2021 (B.1.1.529+R346K); BA.2: hCoV-19/USA/CO-CDPHE-2102544747/2021; BA.2.12.1: hCoV-19/USA/NY-MSHSPSP- PV56475/2022 were obtained, sequenced, and confirmed to be aligned with consensus sequences. BA.4: hCoV-19/USA/MD-HP30386/2022 was obtained, sequenced, and confirmed to be aligned with consensus sequences except containing an additional V3G in the spike protein. mAbs are diluted from 10,000 to 4.88 ng/ml across 12 wells with duplicate rows for each sample. Multiplicity of infection (0.01) for each strain is added to dilution wells and incubated for 1 hour at 37°C. Virus and mAb are added to 96-well plates of Vero-TMPRSS2 cells and incubated at 37°C in 5% CO2 incubator for 72 h (wild-type) and 96 h (Omicron subvariants), after which plaques are read for each row. The first mAb dilution to show cytopathic effects was reported as the minimum mAb concentration required to neutralize >99% of the concentration of SARS-CoV-2 tested (neut99) (27).

### Amino acid conservation calculation

The score of amino acid conservation (%) is calculated based on the data downloaded from COVID-19 Viral Genome Analysis Pipe Line website (https://cov.lanl.gov/components/sequence/COV/int_sites_tbls.comp?t=2).

### *In vivo* animal study

Syrian golden hamster studies were conducted at U.S. Army Medical Research Institute of Infectious Diseases (Fort Detrick, MD). Briefly, PBS or the 1:1 combination of saline, 10 mg/kg or 50 mg/kg of amubarvimab and romlusevimab in 1:1 combination, were administered intraperitoneally (i.p.) to Syrian hamsters 24 hours before or 8 hours after intranasal (i.n.) instillation of 100,000 PFU of SARS-CoV-2 USA-WA1/2020 (US Centers for Disease Control and Prevention, Atlanta, GA) isolate in 100 uL of PBS. A group of animals with mock exposure was also included as weight controls. After challenge, the animals were weighed and observed daily until the end of the study. On day 3 and day 7, lung samples were collected to determine the viral load by plaque assay and by quantitative RT-PCR assay based on E gene subgenomic RNA. Briefly, lung samples were homogenized in cell culture medium, clarified by centrifugation, and supernatants removed. Plaque titrations were performed on serial dilutions of the clarified homogenate to quantify infectious virus in PFU as previously reported (28). Total RNA was extracted from clarified lung homogenates in Trizol™ LS, and the viral RNA was quantified using E gene subgenomic RT-PCR assay as previously described (29). The histopathology of the lung was evaluated based on lung tissue sections fixed with 10% neutral buffered formalin, paraffin embedded, and then hematoxylin and eosin (H&E) stained (30). Slides were visualized and scored for the degree of interstitial pneumonia based on the percent of tissue affected as 1 = 0-25%, 2 = 26-50%, 3 = 51-75%, and 4 = 76-100%. One animal was excluded from both 10 mg/kg and 50 mg/kg groups in the prophylactic setting due to a failed infusion of mAbs based on Day 3 Human IgG levels in serum by ELISA or PsVNA50 titer in the pseudovirus neutralization assay.

## Results

### Amubarvimab and romlusevimab non-competitively bind to RBDwith modified Fc

**Figure 1:**
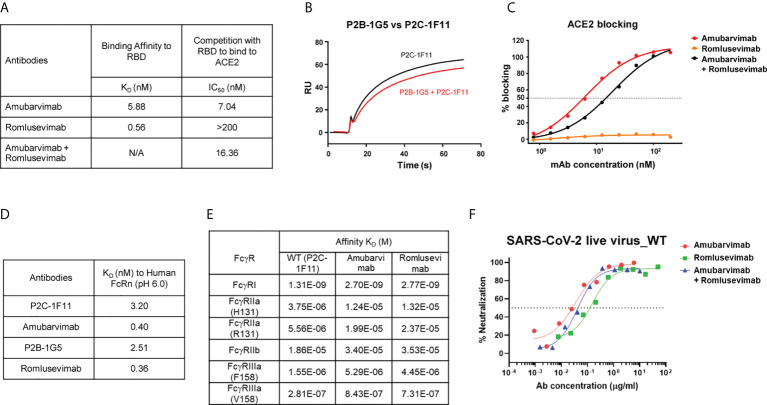
Amubarvimab and romlusevimab non-competitively bind to RBD with modified Fc. **(A)** Amubarvimab and romlusevimab binding affinity measured by SPR to RBD and degree of competition with RBD to bind to ACE2. **(B)** Competition of P2B-1G5 and P2C-1F11, the precursors of amubarvimab and romlusevimab, for binding to RBD measured by SPR. Blocking efficiency was determined by comparison of response units (RU) with and without prior antibody incubation. **(C)** Competition of amubarvimab and romlusevimab together with ACE2 for binding to SARS-CoV-2 RBD. Measurements were taken across a series of mAb concentrations and the resulting nonlinear regression curves were used to calculate IC_50_ values. **(D)** Binding affinity of amubarvimab, romlusevimab and their precursors P2C-1F11 and P2B-1G5 to human FcRn at pH 6.0 measured by SPR. **(E)** Binding affinity of amubarvimab and romlusevimab to human FcγRs measured by SPR. **(F)** Neutralization potency of amubarvimab, romlusevimab, and their 1:1 combination against SARS-CoV-2 wild-type live virus. All data are representative of at least two independent experiments.

The SARS-CoV-2 neutralizing antibodies P2C-1F11 (precursor of amubarvimab) and P2B-1G5 (precursor of romlusevimab) were selected as candidate antibodies for the development of a therapeutic mAb cocktail based on their potent anti-viral activities and distinct competition binding profiles with ACE2 (18, 31, 32). Amubarvimab and romlusevimab exhibited high binding affinity to the RBD with equilibrium dissociation constant (K_D_) values of 5.88 nM and 0.56 nM, respectively (**Figure 1A**). P2C-1F11 exhibited no or minimal competition with P2B-1G5 for the binding to RBD (**Figure 1B**). Furthermore, amubarvimab and the combination of amubarvimab and romlusevimab blocked the binding of human ACE2 receptor to RBD with half-maximal inhibitory concentration (IC_50_) values of 7.04 nM and 16.36 nM, respectively. By contrast, romlusevimab alone did not compete with ACE2 for binding to RBD even at the highest concentration tested (IC_50_ >200 nM) (**Figures 1A, C**), suggesting that these two mAbs are an ideal pair of non-competing mAbs that can bind RBD simultaneously to block SARS-CoV-2 entry.

The YTE substitutions were engineered into the Fc region of the precursor antibodies P2C-1F11 and P2B-1G5 to yield amubarvimab and romlusevimab. These modifications resulted in an approximately 10-fold increase of binding affinity to human neonatal Fc receptor (FcRn) at pH 6.0 compared to precursor control antibodies (**Figure 1D**) and a 2- to 4-fold extended serum half-life (t_1/2_) in cynomolgus monkeys (**Supplementary Figure 1**) and in humans (33). The YTE substitutions also reduced their binding affinities to Fcγ receptors by approximately 3-fold (**Figure 1E**), consistent with the design to reduce effector functions and resulted in minimal antibody-dependent cellular toxicity and undetected antibody-dependent enhancement of viral entry and/or viral replication in cell-culture based assays (data not shown). The neutralizing activity of Fc-engineered mAbs were evaluated against wild-type live virus in microneutralization assays. Amubarvimab and romlusevimab alone or in combination exhibited potent neutralizing activity with mean IC_50_ values of 0.026, 0.156, and 0.047 µg/mL respectively, which were comparable to those of their respective precursor antibodies (**Figure 1F**; **Supplementary Figure 2**).

### *In vitro* selection and characterization of SARS-CoV-2 resistant viruses to amubarvimab and romlusevimab

The monoclonal antibody resistant mutants (MARMs) were selected by serial passage of a replication-competent recombinant VSV-SARS-CoV-2 system in the presence of a single antibody (amubarvimab or romlusevimab) as well as the combination of the two antibodies. Analysis of RBD sequences of selected viruses from two independent experiments revealed two single amino acid changes at position F456 and N460 for amubarvimab, and four R346, N354, L452 and F490 substitutions for romlusevimab. The N460 was identified at the highest frequency in the presence of amubarvimab whereas R346 was for romlusevimab in both passage 1 and 2 (**Table 1**). However, in the presence of combination of amubarvimab and romlusevimab, no reproducible amino acid changes were detected following two passages, indicating that the antibody combination can prevent mutational escape. As expected, all amino acid changes identified in these MARMs were located in the binding sites of amubarvimab or romlusevimab defined by structural determination (19).

**Table 1:** Neutralization of amubarvimab and romlusevimab against pseudovirus encoding amino acid substitutions identified in the escape mutants.

mAbs	Amino acid substitutions in the variants tested	Mutant variants (%)*		Average fold change in IC_50_ relative to wild-type		
		Passage 1	Passage 2	Amubarvimab	Romlusevimab	Amubarvimab + Romlusevimab
Amubarvimab	F456V	30%		>90.0	1.1	6.2
	N460H	50%, 100%	100%, 100%	>115.7	0.9	4.7
Romlusevimab	R346Q	10%, 35%	100%	0.3	>140.6	0.3
	R346W	80%	100%	0.7	>140.6	0.3
	N354D	5%, 15%		1.0	>267.7	0.5
	L452R	35%		1.0	199.9	1.3
	F490S	5%		1.2	>17	n.d.

*: numbers representing results from two independent experiments; undetected (0%) not listed; n.d., not determined.

To evaluate the effect of the amino acid substitutions identified in the MARMs on the susceptibility to neutralization by amubarvimab and romlusevimab, the pseudoviruses carrying these single substitutions were generated and subjected to a microneutralization assay. The F456V and N460H substitutions resulted in greatly reduced susceptibility to amubarvimab neutralization (>90- or >116-fold, respectively) but no detectable effect on romlusevimab alone or in combination with amubarvimab (**Table 1**). The pseudoviruses bearing R346Q/W, N354D, L452R, F490S substitutions exhibited substantial resistance to romlusevimab, with respective estimated IC_50_ of >140-fold, >268-fold, 200-fold, or >17-fold relative to the parental strain. No detectable impact, however, was found on amubarvimab. Importantly, all variants evaluated showed no or up to 6-fold reduction to neutralization by the combination of amubarvimab and romlusevimab (**Table 1**), supporting the choice of the two antibodies for the development of effective therapeutics to overcome resistance.

### Characterization the impact of natural polymorphisms in the epitopes of amubarvimab and romlusevimab

**Figure 2:**
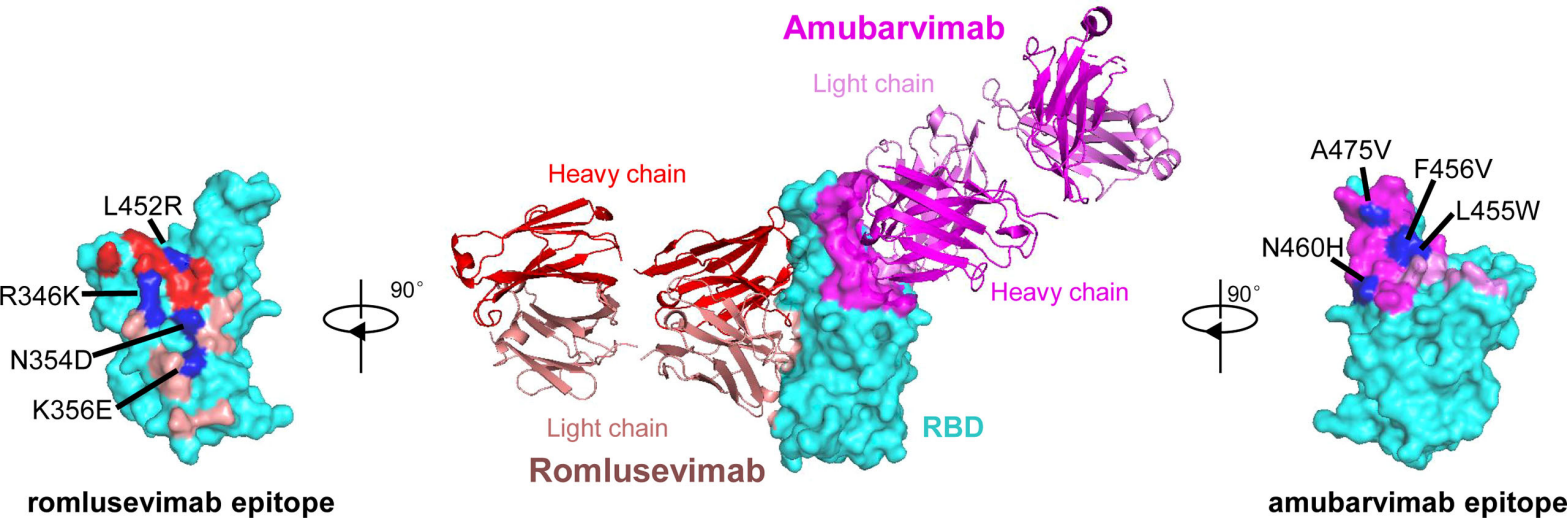
Co-crystal structure of SARS-CoV-2-RBD/amubarvimab/romlusevimab. Amubarvimab and romlusevimab simultaneously bind to distinct, nonoverlapping epitopes on the RBD of spike protein. A side-view depiction shows cartoon representations of amubarvimab (magenta) and romlusevimab (red) together with RBD (cyan) in surface representation based on co-crystal structure of amubarvimab and romlusevimab Fabs with RBD. Romlusevimab epitope (red) and amubarvimab epitope (magenta) with mutation sites of impact on neutralization IC_50_ over approximately 100-fold are marked in blue.

To investigate the epitopes of romlusevimab, we determined the crystal structures of the Fab fragments of amubarvimab and romlusevimab in complex with SARS-CoV-2 RBD at a resolution of 4.01 Å (**Figure 2**). Consistent with our previous report (19), amubarvimab exclusively binds to the RBM region, extensively overlapping with the ACE2 binding site. By contrast, romlusevimab bound to RBD from an opposite side of RBD and has no clash with amubarvimab (**Figure 2**), consistent with the noncompetitive feature of amubarvimab and romlusevimab. The structure analysis revealed a discontinuous epitope of romlusevimab that contained 18 amino acid residues largely located in the core domain of RBD with only one residue shared with the ACE2-binding site. These residues clustered within two regions spanning amino acids 334-360 in the core region and 444-466 in the RBM of RBD, with no overlap with amubarvimab binding sites (**Figure 2**).

To determine the impact of natural polymorphisms identified in the epitopes of amubarvimab and romlusevimab, pseudoviruses carrying 20 of 23 and 16 of 18 substitutions were successfully generated and tested for their susceptibilities to amubarvimab and romlusevimab individually or in combination. Among all the substitutions tested, those confined to 4 positions resulted in reduced susceptibility to amubarvimab alone with IC_50_ increased more than ~100-fold (**Supplementary Table 1**; **Figure 2**). Likewise, mutant pseudoviruses containing contact residues at 4 positions in the epitope of romlusevimab exhibited 100-fold or higher reduced susceptibility to romlusevimab neutralization (**Supplementary Table 2**; **Figure 2**). The combination of amubarvimab and romlusevimab maintained activity against 50 of 61 mutant pseudoviruses tested and had moderate 5- to 25-fold reduced activity against the remaining 10 pseudoviruses (**Supplementary Table 1**; **Supplementary Table 2**). Notably, these 10 amino acid substitutions were only detected in low frequencies, i.e., <0.1% out of 2,731,077 GISAID sequences analyzed as of May 31st, 2022.

### Low cross-resistance potential of amubarvimab and romlusevimab to other mAb therapeutics

We next investigated to what extent the cross-resistance occurred to 21 single substitutions in the spike protein that confer reduced susceptibility to the mAbs authorized by EUA to treat COVID-19. To this end, pseudoviruses bearing these substitutions were generated and subjected to neutralization by amubarvimab and romlusevimab combination in microneutralization assays. Of 21 mutant pseudoviruses tested, 17 remained sensitive to amubarvimab and romlusevimab combination with their IC_50_ maintained within 3-fold changes relative to that of wild-type. Only four mutant pseudoviruses containing K417E, L455F, F486V, or Q493K substitutions resulted in considerable reduction of neutralizing activity of amubarvimab and romlusevimab combination (~6- to 25-fold changes in IC_50_s) (**Table 2**).

**Table 2:** Neutralization of amubarvimab and romlusevimab against variant pseudoviruses conferring reduced susceptibility to authorized mAbs.

Amino acid substitution in tested variant	mAb with reduced susceptibility	Average Fold change in IC_50_ relative to wild-type		
		Amubarvimab	Romlusevimab	Amubarvimab + Romlusevimab
P337L	sotrovimab	1.0	4.1	2.0
P337R	sotrovimab	0.7	1.8	0.8
E340A	sotrovimab	0.6	0.5	0.4
E340K	sotrovimab	0.5	0.5	0.6
K417E	casirivimab	31.9	0.3	5.5
K417N	casirivimab etesevimab	3.1	0.5	2.2
N439K	imdevimab	0.9	0.8	1.3
K444Q	imdevimab	0.6	0.6	0.8
V445A	imdevimab	0.9	0.8	1.0
G446V	imdevimab	1.0	0.4	0.6
N450D	imdevimab	0.6	11.0	0.9
Y453F	casirivimab	1.1	1.0	1.2
L455F	casirivimab	477.3	1.6	24.5
E484K	bamlanivimab casirivimab	1.6	3.6	2.7
E484Q	bamlanivimab	1.5	2.6	1.6
F486V	casirivimab	52.8	0.9	7.3
F490S	bamlanivimab	1.3	134.9	1.1
Q493E	casirivimab	1.8	0.8	0.9
Q493K	bamlanivimab, casirivimab etesevimab	20.0	1.0	6.5
S494P	bamlanivimab, casirivimab	0.7	0.7	0.6
P499S	imdevimab	0.8	0.8	0.7

### Amubarvimab and romlusevimab demonstrate broad neutralization against most SARS-CoV-2 variants *in vitro*

**Figure 3:**
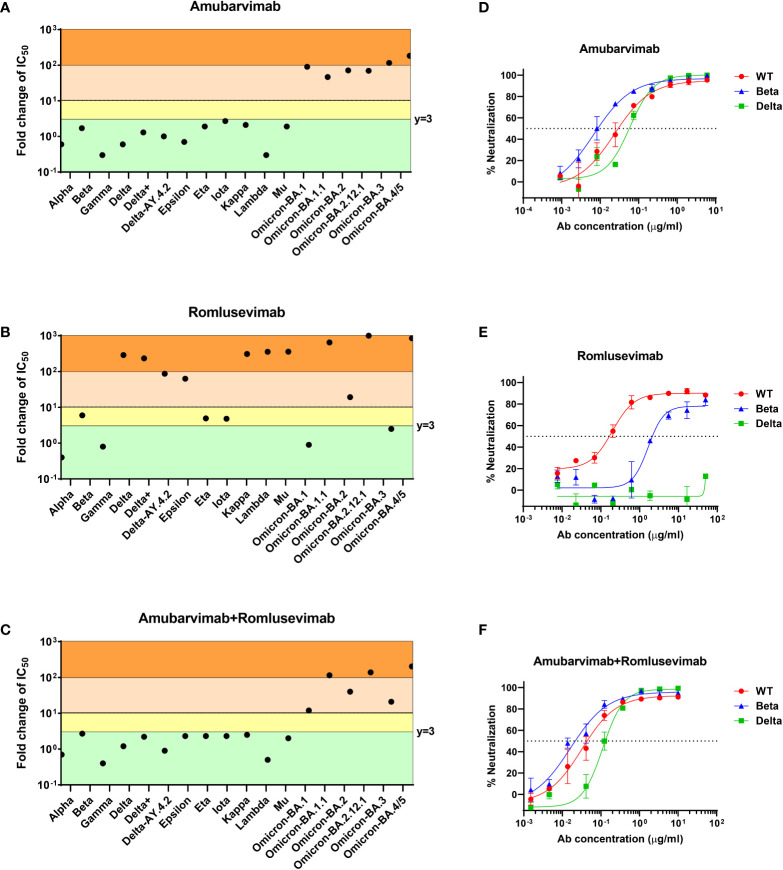
Amubarvimab, romlusevimab and amubarvimab+romlusevimab neutralize SARS-CoV-2 VOCs/VOIs *in vitro*. **(A–C)** Neutralization potency of amubarvimab, romlusevimab, and their 1:1 combination against SARS-CoV-2 VOCs/VOIs. Data shown represents fold-change in neutralization potencies (IC_50_) of amubarvimab **(A)**, romlusevimab **(B)** and amubarvimab+romlusevimab **(C)** against the past and present circulating VOCs/VOIs compared with the D614G wild-type pseudotyped VLPs. **(D–F)** *In vitro* neutralization of wild-type, Beta and Delta authentic live virus with amubarvimab **(D)**, romlusevimab **(E)**, and amubarvimab and romlusevimab together **(F)**. Results are representative of at least two independent experiments.

We further evaluated combination of amubarvimab and romlusevimab against wide varieties of VOCs/VOIs emerged during pandemic. In the pre-Omicron era, amubarvimab or the combination of amubarvimab and romlusevimab maintained neutralizing activities against all pseudovirus expressing the spike of Alpha (B.1.1.7), Beta (B.1.351), Gamma (B.1.1.248/P.1), and Delta (B.1.617.2), although romlusevimab alone showed significantly reduced activity against VOCs/VOIs containing R346K, L452R/Q, or F490S single substitutions, which is consistent to its MARM profile (**Figures 3A-C**).

The impact of the highly immune evasive B.1.1.529 (Omicron) variants on amubarvimab and romlusevimab was also assessed using the pseudovirus system. Significant activity reductions in susceptibility of these sub-lineage variants tested (BA.1, BA.1.1, BA.2, BA.2.12.1, BA.3, and BA.4/5) were observed to the neutralization by amubarvimab (**Figure 3A**; **Supplementary Table 3**). However, romlusevimab maintained activity against BA.1 and BA.3 (<3-fold change in IC_50_s), showed moderate activity reduction against BA.2 (~20-fold change in IC_50_), and significant activity reduction against BA.1.1, BA.2.12.1, and BA.4/5 likely due to the additional R346K or L452Q/R identified in these subvariants (**Figure 3B** and **Supplementary Table 3**). Compared to wild-type pseudovirus, the combination of amubarvimab and romlusevimab exhibited varying activity reduction in IC_50_s (ranging from 10- to 200-fold) against Omicron subvariants (**Figure 3C**; **Supplementary Table 3**), consistent with results generated in other independent studies (15–17, 34–36).

We next measured neutralizing activity of amubarvimab and romlusevimab against authentic live virus whenever possible including recently prevalent Omicron subvariant isolates. Amubarvimab alone or amubarvimab and romlusevimab in combination revealed similar levels of neutralizing activity against live virus Alpha, Beta and Delta compared to wild-type, although romlusevimab showed moderate to significant reduction of activities against these variants (7 to >320-fold change in IC_50_s) (**Table 3** and **Figures 3D-F**). Neutralizing activity against Omicron sub-lineage live virus was also evaluated using an endpoint assay in which the first mAb dilution to show cytopathic effects was reported as the minimum concentration required to neutralize >99% of the SARS-CoV-2 tested (Neut99). Based on this assay, there were minor to moderate reductions in neutralizing activity of the amubarvimab and romlusevimab combination against BA.1, BA.2, BA.2.12.1, and BA.4/5 (ranging from 3- to 16-fold) compared to wild-type live virus, with Neut99s ranging between 0.47 and 2.50 µg/mL, respectively. The activity of amubarvimab and romlusevimab in combination against BA.1.1 decreased >64-fold to a Neut99 of >10.00 μg/mL (**Table 3**). Altogether, amubarvimab and romlusevimab demonstrate broad neutralizing activity against a wide range of SARS-CoV-2 variants including various Omicron subvariants.

**Table 3:** Amubarvimab and romlusevimab effectively neutralize most live viruses tested.

Lineage	WHO naming convention	Key amino acid substitutions in RBD	Amubarvimab	Romlusevimab	Amubarvimab + Romlusevimab
**Average fold-change in IC_50_ relative to SARS-CoV-2 wild-type USA-WA1/2020**					
USA-WA1/2020	NA	None	1.0	1.0	1.0
B.1.1.7-CA	Alpha	N501Y	0.5	0.5	0.4
B.1.1.7-PHE	Alpha	N501Y	0.2	0.3	0.2
B.1.351	Beta	E484K, N501Y	0.7	7.0	1.4
**Average fold-change in IC_50_ relative to SARS-CoV-2 wild-type Beta/Shenzhen/SZTH-003/2020**					
Beta/Shenzhen/SZTH-003/2020	NA	None	1.0	1.0	1.0
B.1.351	Beta	K417N, E484K, N501Y	0.4	15.3	0.4
B.1.617.2	Delta	L452R, T478K	2.1	>320.5	2.9
**Neutralization data of amubarvimab and romlusevimab together against live viruses of Omicron sub-lineages and wild-type WA1/2020**					
SARS-CoV-2	Sub-lineages	Key amino acid substitutions in RBD		Neut99 of amub. + roml. (μg/ml)	Fold-change in Neut99 relative to wild-type
Wild-type	WA1/2020 (CDC)	None		0.16	1.0
B.1.1.529	BA.1	G339D, S371L, S373P, S375F, K417N, N440K, G446S, S477N, T478K, E484A, Q493R, G496S, Q498R, N501Y, Y505H		0.63	4.0
	BA.1.1	G339D, R346K, S371L, S373P, S375F, K417N, N440K, G446S, S477N, T478K, E484A, Q493R, G496S, Q498R, N501Y, Y505H		>10.00	>64.0
	BA.2	G339D, S371F, S373P, S375F, T376A, D405N, R408S, K417N, N440K, S477N, T478K, E484A, Q493R, Q498R, N501Y, Y505H		2.50	16.0
	BA.2.12.1	G339D, S371F, S373P, S375F, T376A, D405N, R408S, K417N, N440K, L452Q, S477N, T478K, E484A, Q493R, Q498R, N501Y, Y505H		0.47	2.9
	BA.4/5	G339D, S371F, S373P, S375F, T376A, D405N, R408S, K417N, N440K, L452R, S477N, T478K, E484A, F486V, Q498R, N501Y, Y505H		0.94	5.9

### Amubarvimab and romlusevimab display potent activity against SARS-CoV-2 *in vivo*

**Figure 4:**
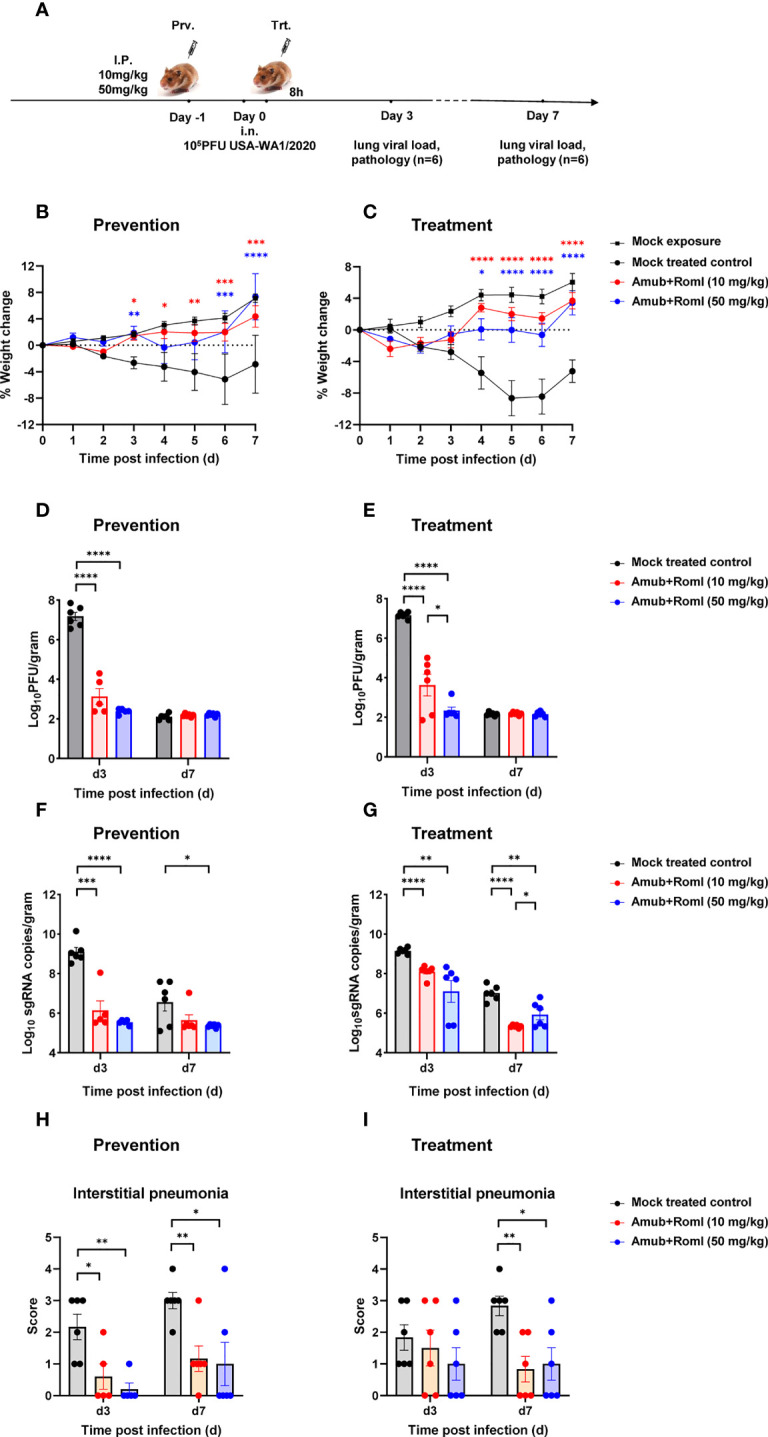
Amubarvimab and Romlusevimab together show *in vivo* efficacy in a hamster model of SARS-CoV-2 infection. **(A)** Overview of *in vivo* study design. mAb or PBS was injected i.p. 24 hr before **(B, D, F, H)** or 8 hr after **(C, E, G, I)** intranasal challenge with 100,000 PFU live virus on day 0. **(B, C)** Body weigh change of Syrian hamsters relative to day 0. Animals were weighed daily, and the percent of weight change was plotted. Symbols represent mean ± standard error of the mean (SEM). **(D, E)** Viral burden reflected by PFU in the lung tissues. PFU was measured and normalized with weights. Bars represent the geometric means ± SEM in PFU per gram. **(F, G)** Viral burden reflected by subgenomic (sg) RNA of virus E gene in the lung tissues. Quantitative PCR was performed to measure the copies of sgRNA of virus E gene in lung tissue homogenates. The bars represent the geometric mean of subgenomic RNA copies per gram ± SEM. **(H, I)** Pathology scores of lung sections. A pathology score was assigned by board-certified veterinary pathologist based on histologic findings on H&E-stained lung sections. Data are presented as mean + SEM. Significant differences relative to the comparative group using unpaired t-test are shown as asterisk: *p < 0.05, **p < 0.01, ***p < 0.001, and ****p < 0.0001 (unpaired two-tailed Student’s *t*-test).

To assess whether the potency against SARS-CoV-2 exhibited by amubarvimab and romlusevimab *in vitro* can be translated to protectivity *in vivo*, weight-based doses of amubarvimab and romlusevimab combination were administered to Syrian golden hamsters through intraperitoneal injection. One day later, animals were intranasally challenged with 10^5^ plaque forming unit (PFU) SARS-CoV-2 live virus (USA-WA1/2020) and monitored for survival and body weight change. Lungs were harvested at day 3 and day 7 to determine viral load and pathology scores (**Figure 4A**). Compared to the untreated control animals, hamsters receiving either the 10 mg/kg or 50 mg/kg dose of 1: 1 amubarvimab and romlusevimab exhibited no body weight loss during the 7-day observation period (**Figure 4B**). Furthermore, we tested therapeutic potential of amubarvimab and romlusevimab combination in the same animal model by administrating these antibodies into animals 8-hours post infection. Treated animals showed minor weight loss (<3%) at days 1 and 2 but quickly regained body weight day 3 compared to control animals (**Figure 4C**).

The viral load in the lung tissues collected from each group on day 3 and 7 post infection was determined by plaque forming assay and RT-PCR. Consistent with body weight changes, administration of 10 mg/kg of amubarvimab and romlusevimab in either a prophylactic or therapeutic setting resulted in >3.5 logs viral load reduction in PFU on day 3 as compared to the mock treated control group. Higher dose of 50 mg/kg resulted in >4.5 logs viral load reduction in PFU on day 3 in both settings, demonstrating a dose-dependent response. By day 7 post infection, the infection was naturally resolved and no significant difference in lung PFU was found between treated and mock treated groups (**Figures 4D, E**; **Supplementary Table 4**). Similarly, administration of 10 mg/kg of amubarvimab and romlusevimab reduced lung sgRNA copies by approximately 3 logs in the prophylactic or by approximately 1 log in the therapeutic setting. In the higher dose of 50 mg/kg treated animals, lung sgRNA copies reduced by approximately 3.5 logs in the prophylactic setting or by approximately 2 logs in therapeutic setting on day 3 after infection, further supporting a dose-dependent response (**Figure 4F, G**; **Supplementary Table 4**).

Consistent with the reduced viral loads in the lung, prophylactic administration of a 10 mg/kg or 50 mg/kg dose of amubarvimab and romlusevimab combination can also significantly alleviate interstitial pneumonia, demonstrated by substantially reduced pathology scores on day 3 and day 7 after infection (**Figure 4H**). Similar findings were observed on day 7 in the therapeutic setting (**Figure 4I**). Collectively, these *in vivo* animal studies further reinforce the potent efficacy of amubarvimab and romlusevimab combination in protecting animals from infection in both prophylactic and therapeutic settings.

## Discussion

In this study, amubarvimab and romlusevimab demonstrated high-affinity binding to distinct non-competing epitopes on the RBD of SARS-CoV-2 spike protein and effective neutralization of wild-type SARS-CoV-2 live virus *in vitro*. In addition, this mAb combination preserved activity against most MARMs of mAbs previously authorized for emergency use, as well as most single-mutant variants in the epitope region, therefore building a high barrier to resistance. Consistently, these two mAbs in combination retained activity against most circulating VOCs/VOIs tested using pseudoviruses and live virus isolates, proving to be an ideal mAb pair to control viral spread and prevent resistance. In a Syrian golden hamster infection model, animals receiving amubarvimab and romlusevimab together pre- or post-infection with wild-type SARS-CoV-2 showed less weight loss, significantly decreased viral load in the lungs, and reduced lung pathology compared to controls. The YTE modification on amubarvimab and romlusevimab not only reduced binding to FcγRs as desired but also increased recirculation through the enhanced binding to FcRn resulted in an extended mAb half-life in a cynomolgus monkey PK study. Therefore, based on the *in vitro* neutralization activity and PK analysis, a clinical dose of 1000 mg each for amubarvimab and romlusevimab was selected to treat non-hospitalized adults with mild to moderate COVID-19 at high risk of progression to severe disease in a Phase 2/3 study (ACTIV-2/A5401) and positive clinical outcomes were obtained showing this mAb combination significantly reduced the risk of hospitalization and death among COVID-19 outpatients at high risk (24). Taken together, the combination of amubarvimab and romlusevimab demonstrated potent therapeutic efficacy in both preclinical and clinical studies, adding another pair of mAb options to the current antibody therapeutics pool to fight against COVID-19.

Overall, the amubarvimab and romlusevimab combination has a good breadth of coverage against SARS-CoV-2 variants. Firstly, most predicted contact residues in the amubarvimab and romlusevimab epitope regions remain highly conserved among available sequences of circulating virus with ≥99.9% conservation as of May 31^st^, 2022. Secondly, the remaining several contact residues including R346 (76.12%), K417 (23.22%), L452 (77.63%), S477 (21.11%), Q493 (21.32%), and Y505 (21.28%) showed reduced conservation because these mutations appear in VOC spike proteins such as Beta (K417), Gamma (K417), Omicron 4 subvariants BA.1, BA.1.1, BA.2, BA.3 (K417, S477, Q493, Y505), Mu/BA.1.1 (R346), and Delta (L452), suggesting mutations arose at these sites under immune selection pressure (17, 37). However, the amubarvimab and romlusevimab combination effectively neutralized pseudotyped variants encoding single amino acid substitutions at these sites due to the complementary neutralizing effects of the mAbs when one mAb was affected. Thirdly, amubarvimab and romlusevimab together retained effective antiviral activity against most SARS-CoV-2 VOCs/VOIs in both pseudovirus and live virus *in vitro* microneutralization assays. Intriguingly, the variants most refractory to therapeutic antibodies and sera neutralization from convalescent and vaccinated individuals, such as Beta and BA.1 (17, 37, 38), are fully susceptible to the combination of amubarvimab and romlusevimab, indicating the breath of their coverage. This is further supported by a recent *in vivo* study that a single intraperitoneal injection of amubarvimab and romlusevimab together at 10 mg/kg can effectively protect K18-hACE2 transgenic mice from BA.1 challenge, showing significant viral load reduction in the lungs and reduced lung pathology compared to the controls (16). Lastly, the breadth of coverage is further supported by the clinical data from the Phase 2/3 ACTIV-2 study using 1000 mg each of amubarvimab and romlusevimab concomitantly administered to treat outpatients with mild to moderate COVID-19. In this study, significant clinical improvements were observed in participants infected with Alpha, Beta, Gamma, or Delta (~20% of participants) variants (Evering et al. in preparation), showing that patients infected with these variants are susceptible to the combination of amubarvimab and romlusevimab, consistent with *in vitro* analysis. This also indicates that *in vitro* neutralizing activity of amubarvimab and romlusevimab is a good predictor for the *in vivo* efficacy of the combination, which was also observed with AZD7442 (39).

As of July 2022, BA.4/5 is the most prevalent SARS-CoV-2 variant worldwide. BA.4/5 exhibited reduced susceptibility (6-fold change in Neut99 relative to wild-type live virus) to the combination of amubarvimab and romlusevimab in *in vitro* live virus microneutralization assays. To estimate the impact of the BA.4/5 variant on the presumptive clinical efficacy of the antibody combination, we performed a detailed analysis and prediction based on BA.4/5 live virus neutralization result and PK modeling generated from the interim human population PK analysis performed as part of the ACTIV-2/A5401 study of non-hospitalized COVID-19 patients. The PK model predicts median serum concentrations at day 14 of 86.8 and 81.9 μg/mL following IV infusion of 1000 mg each of amubarvimab and romlusevimab, respectively, and the corresponding predicted concentrations at day 28 are 56.3 and 68.6 μg/mL (unpublished). The estimated total serum mAb concentrations at day 14 and day 28 post-infusion are >170- and >120-fold of the Neut99 (0.94 μg/mL) of amubarvimab and romlusevimab combination against live virus isolate BA.4/5. Therefore, based on the PK data and these cell-based neutralization assay results using authentic viruses, the amubarvimab and romlusevimab total serum exposures are anticipated to be effective *in vivo* against current circulating Omicron subvariant BA.4/5 during the commonly recognized 2-week treatment window post administration. Adequate therapeutic exposures are expected to persist for a minimum of 4 weeks, or longer. Nevertheless, further pressure on the clinical regimen is possible due to the continuing mutation of SARS-CoV-2, requiring continued vigilant surveillance.

Taken together, the *in vitro* and *in vivo* data suggest that amubarvimab and romlusevimab are a pair of well-chosen non-competing mAbs with superior efficacy, extensive breadth of coverage, prolonged half-life, and high serum exposure during the treatment window, warranting them another noteworthy drug to fight against COVID-19.

## Data availability statement

The original contributions presented in the study are included in the article/**Supplementary Material**. Further inquiries can be directed to the corresponding authors.

## Ethics statement

The animal study was reviewed and approved by U.S. Army Medical Research Institute of Infectious Diseases (USAMRIID), Fort Detrick, MD, USA.

## Author contributions

YJ, QZa, LC, JG, RW, MeF, EM, PC, JM, RZ, MH, MaF, ZZ, XW, JH, LZ and QZu designed the study and interpreted the data; QZa, LC, JG, RW, MeF, EM, PC, RZ, HH, LB performed the experiments; YJ, RW, LC, QZa, EM, JM and QZu analyzed the data; CL performed statistical analysis. MH, MaF, ZZ, JH, XW, LZ supervised the study. YJ, LZ and QZu wrote the manuscript. All authors read, edited and approved the final manuscript.

## Funding

This work was supported by Brii Biosciences and TSB Therapeutics. MF, HH and LB are funded by DHHS/ASPR-BARDA #ASPR-20-01495. ZZ and LZ are funded by the National Key Plan for Scientific Research and Development of China (2021YFC0864500), LZ is also funded by the National Natural Science Foundation (9216920007) whereas ZZ by the Science and Technology Innovation Committee of Shenzhen Municipality (JSGG20200207155251653, JSGG20200807171401008).

## Acknowledgments

We thank Carol Weiss and her laboratory for providing part of the pseudovirus neutralization data encoding single amino acid substitutions or all substitutions in the spike protein of circulating VOC/VOIs at the Center for Biologics Evaluation and Research, US Food and Drug Administration (Silver Spring, Maryland USA) under agreement with NIAID for comparative profiling of nAb products for COVID-19. We thank Chasity Andrews from Brii Biosciences for her assistance of editing the manuscript. We thank David Margolis for his critical review of this manuscript.

## Conflict of interest

YJ, JM, CL and QZ are employees of Brii Biosciences. MF receives funding from National Institute of Allergy and Infectious Diseases (NIAID), Biomedical Advanced Research and Development Authority (BARDA), Defense Advanced Research Projects Agency (DARPA), Gates Foundation, Aikido Pharma, Emergent, Astrazeneca, Novavax, Regeneron, and the CDC, outside the submitted work. MF received royalties/licenses from Aikido Pharma for antiviral drug patent licensing, consulting fees from Aikido Pharma, Observatory Group, for consulting for COVID-19, and participation on Scientific Advisory Board for Aikido Pharma, outside the submitted work.

The remaining authors declare that the research was conducted in the absence of of any commercial or financial relationships that could be construed as a potential conflict of interest.

The authors declare that this study received funding from Brii Biosciences. The funder had the following involvement with the study: Study design, data analysis, and manuscript preparation.

## Publisher’s note

All claims expressed in this article are solely those of the authors and do not necessarily represent those of their affiliated organizations, or those of the publisher, the editors and the reviewers. Any product that may be evaluated in this article, or claim that may be made by its manufacturer, is not guaranteed or endorsed by the publisher.

## Abbreviations

IC_50_, half-maximal inhibitory concentration; mAb, monoclonal antibody; MARM, monoclonal antibody resistant mutant; PFU, plaque forming unit; RBD, receptor binding domain; RBM, receptor binding motif; SARS-CoV-2, severe acute respiratory syndrome coronavirus 2; VOC, variant of concern; VOI, variant of interest; YTE, amino acid M252Y/S254T/T256E substitutions located in the fragment crystallizable domain of antibody.
