# Supplementary material for: Preclinical characterization of amubarvimab and romlusevimab, a pair of non-competing neutralizing monoclonal antibody cocktail, against SARS-CoV-2
Source: Front Immunol. 2022 Sep 14;13:980435. doi: 10.3389/fimmu.2022.980435 (PMC9518701; doi:10.3389/fimmu.2022.980435)
Supplement: Supplementary file 1 [file Table_1.docx]

**Supplementary Table 1 Neutralization of amubarvimab and romlusevimab against pseudo-type variants containing substitution in the binding site of amubarvimab**

| Amino acid position | Consensus sequence | Frequency of conservation* | Variants identified | Amino acid substitution tested | Average Fold change in IC_50_ relative to wild-type | | |
| --- | --- | --- | --- | --- | --- | --- | --- |
|  |  |  |  |  | Amubarvimab | Romlusevimab | Amubarvimab + Romlusevimab |
| 403 | R | 99.99% | K, S, I, -, G, X | R403K | n.d. | | |
|  |  |  |  | R403S | 0.7 | 1.5 | 0.6 |
| 415 | T | 99.95% | N, S, A, K, I, P, X | T415N | 1.2 | 2.8 | 1.1 |
| 416 | G | 99.99% | E, R, $, A, X, # | G416R | n.d. | | |
| 417 | K | 23.22% | K, T, I, H, R, S, Y, M, Q, X, # | K417E | 31.9 | 0.3 | 5.5 |
|  |  |  |  | K417N | 3.2 | 0.5 | 2.2 |
|  |  |  |  | K417R | 0.7 | 1.0 | 0.6 |
|  |  |  |  | K417T | 1.1 | 0.4 | 1.1 |
| 420 | D | 99.99% | N, G, A, Y, X | D420N | 25.3 | 4.8 | 10.4 |
| 421 | Y | 99.99% | F, C, D, N, X | Y421F | 1.4 | 1.2 | 0.9 |
| 453 | Y | 99.99% | F, H, C, N, R, S, X | Y453F | 1.1 | 1.0 | 1.2 |
| 455 | L | 99.94% | F, S, C, W, V, -, A, M, X | L455F | 477.3 | 1.6 | 24.5 |
|  |  |  |  | L455W | >90.0 | 2.3 | 9.5 |
| 456 | F | 99.97% | L, V, -, C, S, Y, X, # | F456V | >90.0 | 1.1 | 6.2 |
| 457 | R | 99.98% | S, K, X, # | R457S | 0.3 | 0.9 | 0.6 |
| 458 | K | 99.98% | N, R, M, E, T, X, # | K458N | 0.9 | 3.3 | 1.2 |
| 460 | N | 99.96% | K, S, Y, D, T, I, H, X, # | N460H | >115.7 | 0.9 | 4.7 |
|  |  |  |  | N460K | 23.6 | 5.6 | 9.0 |
|  |  |  |  | N460S | 11.6 | 1.0 | 2.4 |
| 473 | Y | 100.00% | H, F, X, # | Y473F | 1.0 | 1.0 | 0.9 |
|  |  |  |  | Y473H | 1.5 | 1.8 | 1.5 |
| 474 | Q | 99.99% | H, R, E, K, P, $, X, # | Q474H | 1.0 | 1.6 | 1.2 |
| 475 | A | 99.98% | V, S, T, D, -, P, R, X | A475V | 290.6 | 1.3 | 15.2 |
| 476 | G | 99.97% | S, D, A, C, -, R, V, X | G476S | n.d. | | |
| 477 | S | 21.11% | S, I, G, D, R, K, O, T, -, X | S477N | 0.8 | 1.1 | 1.2 |
|  |  |  |  | S477R | 2.0 | 0.7 | 1.6 |
| 486 | F | 99.89% | V, S, L, I, -, P, X | F486I | 5.5 | 1.5 | 2.1 |
|  |  |  |  | F486K | 32.7 | 1.7 | 3.1 |
|  |  |  |  | F486L | 2.1 | 1.2 | 0.9 |
|  |  |  |  | F486S | 20.2 | 1.1 | 3.1 |
|  |  |  |  | F486V | 52.8 | 0.9 | 7.3 |
| 487 | N | 100.00% | V, -, S, H, K, Y, X | N487D | 15.3 | 1.1 | 3.6 |
| 489 | Y | 99.99% | H, -, F, $, X | Y489H | 71.5 | 1.9 | 11.5 |
| 493 | Q | 21.33% | Q, L, H, E, K, P, G, -, V, X, # | Q493E | 1.8 | 0.8 | 0.9 |
|  |  |  |  | Q493H | 1.4 | 1.0 | 1.4 |
|  |  |  |  | Q493K | 20.0 | 1.0 | 6.5 |
|  |  |  |  | Q493L | 1.1 | 1.3 | 0.8 |
|  |  |  |  | Q493R | 10.2 | 1.3 | 2.8 |
| 502 | G | 100.00% | -, C, V, A, E, Y, D, N, H, L, R, S, X, # | G502S | 0.3 | 1.9 | 0.8 |
| 505 | Y | 21.28% | Y, -, F, L, X, # | Y505H | 0.8 | 1.0 | 1.0 |

*The score of conservation (%) is recorded from COVID-19 Viral Genome Analysis Pipe Line website (https://cov.lanl.gov/components/sequence/COV/int_sites_tbls.comp?t=2) by May 31st, 2022, N=2,731,077; n.d.: not determined; -: deletion; $: a premature stop codon; #: frame shift

**Supplementary Table 2 Neutralization of amubarvimab and romlusevimab against pseudo-type variants containing substitution in the binding site of romlusevimab**

| Amino acid position | Consensus sequence | Frequency of conservation* | Variants identified | Amino acid substitution tested | Average Fold change in IC_50_ relative to wild-type | | |
| --- | --- | --- | --- | --- | --- | --- | --- |
|  |  |  |  |  | Amubarvimab | Romlusevimab | Amubarvimab + Romlusevimab |
| 334 | N | 100.00% | K, S, O, Y, D, -, T, X, # | N334K | 1.3 | 3.8 | 2.0 |
| 337 | P | 99.98% | S, L, R, H, T, N, K, -, I, X, # | P337L | 1.0 | 4.1 | 2.0 |
|  |  |  |  | P337R | 0.7 | 1.8 | 0.8 |
| 340 | E | 99.95% | K, D, A, V, Q, G, P, S, -, H, N, X | E340A | 0.6 | 0.5 | 0.4 |
|  |  |  |  | E340K | 0.5 | 0.5 | 0.6 |
| 345 | T | 99.99% | S, L, I, A, N, X | T345S | 0.6 | 0.5 | 0.5 |
| 346 | R | 76.12% | K, T, S, I, G, -, E, N, A, Q, V, X | R346K | 1.3 | >3448.3 | 1.4 |
|  |  |  |  | R346Q | 0.3 | >140.6 | 0.3 |
|  |  |  |  | R346W | 0.7 | >140.6 | 0.3 |
| 348 | A | 99.97% | S, T, P, V, -, E, X | A348S | 0.7 | 1.3 | 1.0 |
| 349 | S | 99.99% | P, A, -, F, Y, X, # | S349P | 1.0 | 4.3 | 1.4 |
| 351 | Y | 99.98% | -, H, N, F, D, X | Y351F | 2.1 | 3.8 | 1.6 |
| 352 | A | 99.98% | S, V, T, -, D, G, N, X, # | A352S | 1.3 | 1.1 | 0.6 |
| 354 | N | 99.91% | K, D, T, O, H, S, Y, X, # | N354D | 1.0 | >267.7 | 0.5 |
|  |  |  |  | N354K | 1.0 | 0.5 | 1.2 |
| 355 | R | 99.99% | K, S, M, X | R355S | n.d. | | |
| 356 | K | 99.93% | R, T, N, M, Q, E, X | K356E | 1.1 | >714 | 1.0 |
|  |  |  |  | K356R | 0.6 | 6.7 | 1.1 |
| 360 | N | 99.99% | S, K, D, T, Y, X, # | N360Y | n.d. | | |
| 444 | K | 99.95% | R, N, T, O, M, E, I, Q, L, S, X, # | K444E | 0.4 | 1.1 | 0.5 |
|  |  |  |  | K444Q | 0.7 | 0.6 | 0.8 |
| 449 | Y | 99.96% | N, H, D, S, $, -, C, X | Y449S | 0.5 | 5.1 | 1.0 |
| 450 | N | 99.97% | D, K, Y, I, -, H, S, X, # | N450D | 0.6 | 11.0 | 0.9 |
| 452 | L | 77.63% | R, Q, M, W, -, X, # | L452R | 1.0 | 199.9 | 1.3 |
|  |  |  |  | L452Q | 0.8 | 34.8 | 1.0 |
| 466 | R | 99.98% | I, K, T, X | R466I | 0.3 | 14.2 | 0.7 |

*The score of conservation (%) is recorded from COVID-19 Viral Genome Analysis Pipe Line website (https://cov.lanl.gov/components/sequence/COV/int_sites_tbls.comp?t=2) by May 31st, 2022, N=2,731,077; n.d.: not determined; -: deletion; $: a premature stop codon; #: frame shift

**Supplementary Table 3 Neutralization data of amubarvimab and romlusevimab against pseudotyped virus-like particle (VLP) containing spike protein of Omicron subvariants and wild-type (D614G)**

| Omicron Sub-lineages | amubarvimab | romlusevimab | amubarvimab and romlusevimab | |
| --- | --- | --- | --- | --- |
|  | Mean of IC_50_s (µg/ml) | | | Average Fold change in IC50 relative to wild-type (D614G) |
| WT | 0.021 | 0.254 | 0.033 |  |
| BA.1 | 1.901 | 0.230 | 0.389 | 12 |
| WT | 0.017 | 0.154 | 0.012 |  |
| BA.1.1 | 0.787 | >100 | 1.377 | 115 |
| WT | 0.023 | 0.316 | 0.028 |  |
| BA.2 | 1.668 | 6.100 | 1.133 | 40 |
| WT | 0.017 | 0.095 | 0.013 |  |
| BA.2.12.1 | 1.207 | >100 | 1.796 | 138 |
| WT | 0.021 | 0.223 | 0.022 |  |
| BA.3 | 2.416 | 0.559 | 0.455 | 21 |
| WT | 0.007 | 0.059 | 0.012 |  |
| BA.4/5 | 1.269 | >50 | 2.394 | 203 |

Supplementary Table 4 Mean viral load in the lung post infection

|  | | Prevention | | |  | Treatment | | |
| --- | --- | --- | --- | --- | --- | --- | --- | --- |
|  |  | Mock treatment | Amubarvimab + Romlusevimab  cocktail 10 mg/kg | Amubarvimab + Romlusevimab  cocktail 50 mg/kg |  | Mock treatment | Amubarvimab + Romlusevimab  cocktail 10 mg/kg | Amubarvimab +  Romlusevimab  cocktail 50 mg/kg |
| Log10PFU/gram  (Mean ± SEM) | Day3 | 7.18 ±0.21 | 3.14 ±0.40 | 2.39 ±0.06 |  | 7.16 ±0.06 | 3.63 ±0.55 | 2.34 ±0.17 |
|  | Day7 | 2.11 ±0.06 | 2.19 ±0.03 | 2.22 ±0.03 |  | 2.17 ±0.04 | 2.19 ±0.03 | 2.16 ±0.05 |
| Log_10_sgRNA copies/gram  (Mean ± SEM) | Day3 | 9.10 ±0.23 | 6.14 ±0.48 | 5.54 ±0.06 |  | 9.15 ±0.06 | 8.10 ±0.13 | 7.10 ±0.56 |
|  | Day7 | 6.56 ±0.45 | 5.64 ±0.28 | 5.36 ±0.04 |  | 7.01 ±0.16 | 5.34 ±0.03 | 5.93 ±0.26 |
