# Supplementary material for: Preclinical characterization of amubarvimab and romlusevimab, a pair of non-competing neutralizing monoclonal antibody cocktail, against SARS-CoV-2
Source: Front Immunol. 2022 Sep 14;13:980435. doi: 10.3389/fimmu.2022.980435 (PMC9518701; doi:10.3389/fimmu.2022.980435)
Supplement: Supplementary file 2 [file DataSheet_1.pdf]

Supplementary Figure 1

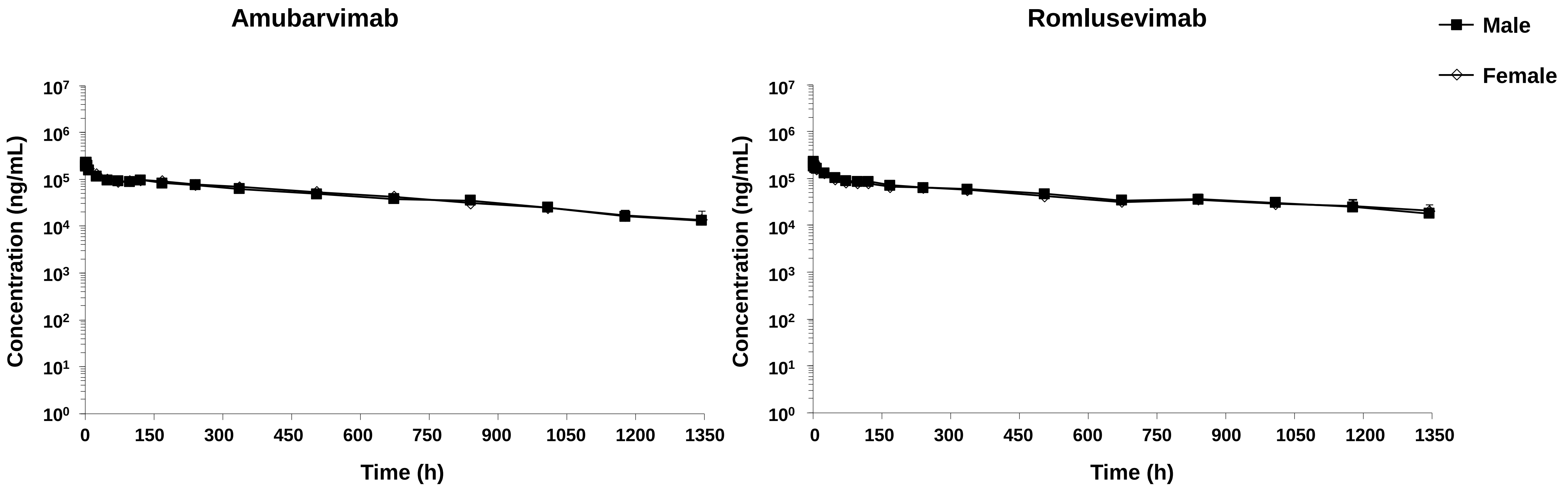

Supplementary Figure 1. Amubarvimab and romlusevimab exhibited extended *in vivo* serum half-life in cynomolgus monkeys. (A B) Mean serum concentration profiles of amubarvimab (A) and romlusevimab (B) in 3 male and female cynomolgus monkeys over 8-weeks following a single intravenous administration of 10 mg/kg.

Supplementary Figure 2

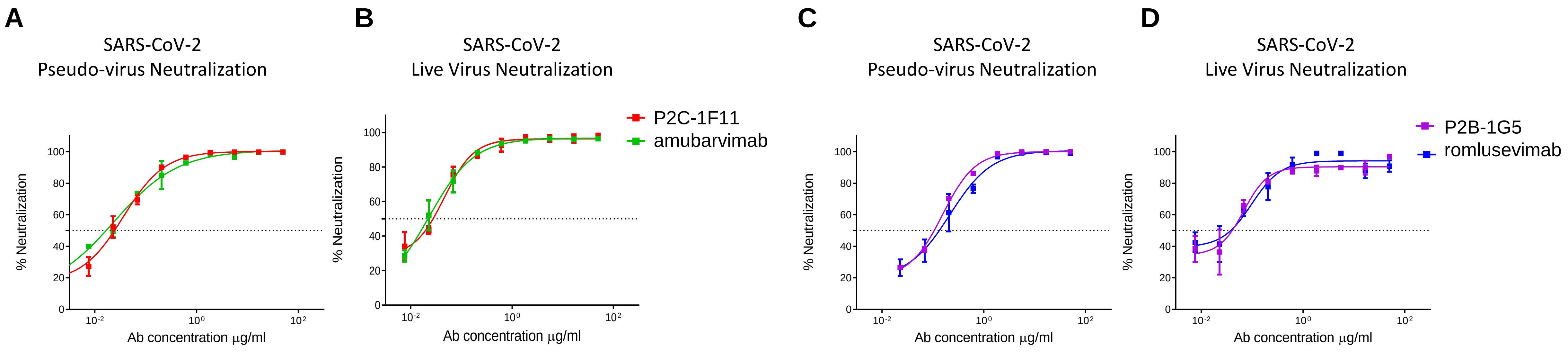

Supplementary Figure 1. The neutralizing activity of amubarvimab and romlusevimab is comparable to their respective unmodified precursor antibodies P2C-1F11 and P2B-1G5. *In vitro* neutralization of wild-type pseudovirus (A, C) or authentic live virus (B, D) with amubarvimab and romlusevimab and their precursor mAbs P2C-1F11 and P2B-1G5. Results are representative of at least two independent experiments.
